# The Role of Robotics in the Laboratory of the 80s

**DOI:** 10.6028/jres.093.016

**Published:** 1988-06-01

**Authors:** James N. Little

**Affiliations:** Zymark Corporation, Zymark Center, Hopkinton, MA 01748

A new technology, robotics, already being used in other fields, is slated to have a major impact in automating operations and procedures performed in chemistry laboratories during the eighties. Introduced only 5 years ago, it has become the fastest growing new technology for the laboratory. An introduction to laboratory robotics, which combines the technologies of chemistry, analytical instrumentation, computers and robotics, will be presented first.

Laboratory automation, once limited to computerized data reduction, can now include sample handling and sample preparation, wet chemistry procedures, and instrumental analysis.

Laboratory robots, utilizing programmable computers, can be easily reprogrammed to do a variety of laboratory procedures and thus, do not require a large quantity of identical, repetitive operations to justify the investment in capital and time.

Examples will be given in automated sample preparation for a variety of trace organic and inorganic analyses.

GC and HPLC applications represent 40% of the current installed base of 1000 laboratory robotic systems. Trace chromatographic assays require many steps (i.e., filtering, extraction, evaporation, etc.) and put tremendous strain on laboratory personnel to maintain acceptable precision levels. Laboratory robotics systems typically improve precision by a factor of 2 or 3 and maintain that precision over extended periods of time. Derivatization, which is routinely used in trace chromatographic analyses, can be easily automated with robotics and eliminates the analyst’s exposure to highly reactive chemicals. Robotics systems can also perform analyses in a serialized mode versus a batch mode. Serialization allows each sample to have the same time history, important in derivatization experiments where possible side reactions can occur causing increased error. Serialization also increases the throughput of samples versus a batch mode.

Automation of wet digestion procedures for trace inorganic analyses has been accomplished using laboratory robotics. Manual procedures require constant attention by an experienced chemist while being exposed to corrosive chemicals and fumes.

Laboratory robotics is finding increased use in microbiological laboratories where a sterile environment must be maintained and care must be taken so that people do not contaminate the experiments.

To improve their utility in the laboratory, robotic systems often must “interface” with standard analytical instruments. This “interface” may include a mechanical handshake to put the sample into the instrument as well as an electronic interface. The electronic interface can be as simple as turning the instrument on and off to completely controlling all parameters on the front panel. Interfaces are available for GC, HPLC, UV/VIS, NMR, titrators, viscometers, computers, ICP, physical testing instruments, etc. Bar code reading systems can also be interfaced to robotic systems allowing sample identification and method selection to be automatically entered.

Laboratory robotics is having an impact on analytical methods, laboratory layout, staffing, type of staffing and work flow. This new level of automation, achieved with laboratory robotics, brings substantial gains in laboratory performance.

